# Aspirin Repurposing in Folate-Decorated Nanoparticles: Another Way to Target Breast Cancer

**DOI:** 10.3389/fmolb.2021.788279

**Published:** 2022-01-28

**Authors:** Fariha Kanwal, Mingming Ma, Muhammad Fayyaz ur Rehman, Fahim-ullah Khan, Shai E. Elizur, Aima Iram Batool, Chi Chiu Wang, Tahira Tabassum, Changrui Lu, Yao Wang

**Affiliations:** ^1^ School of Biomedical Engineering, Med-X Research Institute, Shanghai Jiao Tong University, Shanghai, China; ^2^ Department of Ophthalmology, Shanghai General Hospital, Shanghai Key Laboratory of Ocular Fundus Diseases, National Clinical Research Center for Eye Diseases, Shanghai Engineering Center for Visual Science and Photomedicine, Shanghai Engineering Center for Precise Diagnosis and Treatment of Eye Diseases, Shanghai, China; ^3^ Institute of Chemistry, University of Sargodha, Sargodha, Pakistan; ^4^ Department of Biotechnology, University of Science and Technology, Bannu, Pakistan; ^5^ IVF Unit, Department of Obstetrics and Gynecology, Sheba Medical Center, Tel-Hashomer and Sackler Faculty of Medicine, Tel-Aviv University, Tel-Aviv, Israel; ^6^ Department of Zoology, University of Sargodha, Sargodha, Pakistan; ^7^ Department of Obstetrics and Gynecology, The Chinese University of Hong Kong, Shatin, Hong Kong, China; ^8^ Department of Pathology, Sargodha Medical College, University of Sargodha, Sargodha, Pakistan; ^9^ Department of Chemistry, Chemical Engineering and Biotechnology, Donghua University, Shanghai, China; ^10^ Department of Assisted Reproduction, School of Medicine, Shanghai Ninth People’s Hospital, Shanghai Jiao Tong University, Shanghai, China

**Keywords:** aspirin, nanomedicine, mesoporous silica, breast cancer, nanoparticle

## Abstract

Breast cancer affects more than 1 million women per year worldwide. Through this study, we developed a nanoparticle-based drug delivery system to target breast cancer cells. Aspirin has been found to inhibit thromboembolic diseases with its tumor-preventing activity. As a consequence, it relieves disease symptoms and severity. Here, mesoporous silica nanoparticles (MNPs) have been used to deliver aspirin to the tumor location. MNP-based aspirin in folic acid (F)-conjugated polydopamine (MNP-Asp-PD-PG-F) vehicles are prepared for targeted breast cancer therapy. The vehicle hinges on MNP altered with polymer polyethylene glycol (PG), polydopamine (PD), and F. The delivery vehicle was studied for *in vitro* drug release, cytotoxicity, and breast cancer cell proliferation. F-conjugated drug delivery vehicles let MNPs achieve an elevated targeting efficacy, ideal for cancer therapy. It was also observed that compared to free aspirin, our drug delivery system (MNP-Asp-PD-PG-F) has a higher cytotoxic and antiproliferative effect on breast cancer cells. The drug delivery system can be proposed as a targeted breast cancer therapy that could be further focused on other targeted cancer therapies. Delivering aspirin by the PD-PG-F system on the tumor sites promises a therapeutic potential for breast cancer treatment.

## Introduction

Cancer remains one of the most serious health concerns for human beings and usually requires chemotherapy as a conventional treatment. According to GLOBOCAN, in 2018, 18.1 million new cancer cases arose in a single year with a death toll of 9.6 million (Bray et al., 2018), making cancer the second leading cause of death worldwide. By 2030, cancer will likely claim 27.1 million human lives per year. Breast cancer ranks as the most common cancer in women with over 1 million diagnosed cases annually (Wang et al., 2014). Treatments such as conventional chemotherapy, surgical resection, or radiotherapy have increased its survival rate. However, these approaches require significant improvements. These can be highly aggressive, nonspecific, and systematically toxic because they cannot distinguish between cancer cells and healthy cells, ultimately causing subsequent side effects (Kim et al., 2009; Ceresa et al., 2014; Tao et al., 2015). Bladder cancer therapies, for example, bacillus Calmette–Guérin (BCG), induce CTL-attracting chemokines and suppressive factors and may require some changes in the therapy (Ibrahim et al., 2021).

Since the last decade, nanotechnology has developed more efficient and less toxic therapies to treat cancer. Thus, the current focus shifted to highly stable and efficient nanoparticles (NPs) for cancer therapy since they can modulate the persistent delivery of anticancer agents with lower toxic effects, alter the signal transduction, and regulate the tumor microenvironment for safer and targeted drug delivery (Ferrai, 2005; El-Aassar et al., 2020). This NP technology has received increasing attention because NPs can accumulate and escape the renal filtration process, increasing the retention time of drugs 10 times compared to the free drug (Huo et al., 2013). It can also carry and transport various anticancer agents to specific sites, allowing well-ordered and persistent drug release. With efficient permeability and retaining (EPR) properties, biocompatibility, high uptake at the cellular level and efficient enclosure, and the technology are well suited for cancer therapy (Zhang et al., 2014). A variety of cell-interactive ligands can improve NPs’ targeting and tumoral uptake ability, such as antibodies, nucleic acids, peptides, and other small molecules (Amoozgar and Yeo, 2012; El-Aassar et al., 2021). The folate-PEG decorated Ag/Alg/TMX nanocomposites have shown promising results in breast cancer treatment while selectively inhibiting the MCF-7 cell cycle and inducing an ROS response (Ibrahim et al., 2020).

Among all NPs for cancer therapy, the mesoporous silica nanoparticles (MNPs) contain intricate interior-channeled networks, which capacitates them for higher drug load limits with large surface area, an orderly structure, large pore size, and overall stability (Beck et al., 1992). In addition, large mesoporous channels allow the controlled release of drugs through amorphous pores, improving solubility and drug dissolution (Xu et al., 2013; Riikonen et al., 2018). MNPs show stability in a wide range of recycling and environmental conditions, including extreme temperatures, pH, and high humidity, and are eliminated easily from the human body (Wu et al., 2013). Due to these properties, MNPs became the best choice for cancer therapy (Li et al., 2010; He and Shi, 2014; Chang et al., 2016). They can carry therapy genes or chemotherapeutic agents to the tumor site for targeted cancer drug delivery. The outer surface of MNP requires some coupling agents or reactive linkers to facilitate their activation, blocking the chemotherapeutic agents in the MNPs’ internal pores and controlling the release of drugs (Muhammad et al., 2011; Sardan et al., 2014; Yang and Li, 2014; Florek et al., 2017). Studies show that polydopamine (PD) acts as a common coupling agent to various materials such as ceramics, polymers, metals, and semiconductors (Cong et al., 2014). PD coating adds an adhesive linker on the surface of MNP with enhanced pH sensitivity for the blocking and controlled release of drug molecules. The outer core of MNPs can be adorned with molecules or polymers for controlled drug delivery and release. Folic acid has a strong binding activity to the folate receptor (FR), known as a glycophosphatidylinositol-linked cell surface receptor (Wei et al., 2017). An FR is generally overexpressed on the surface of a variety of human cancerous cells. Various studies have reported that the tight folic acid (FA) binding to its receptor provides a viable FA-conjugated drug delivery system (Cheng et al., 2017a; Cheng et al., 2017b).

Here, we have used MNPs to deliver aspirin to the tumor cell location. Aspirin, also known as acetylsalicylic acid, is a nonsteroidal anti-inflammatory drug typically consumed as an analgesic, antipyretic, anti-inflammatory, and anti-aggregation agent of the platelet (Ng et al., 2019). Studies show that aspirin inhibits the thromboembolic diseases with its tumor-preventing activity (Harrison et al., 2008; Matsuura et al., 2009). Aspirin reduces the cell growth, induces apoptosis and autophagy, and inhibits angiogenesis, which negatively interferes with tumor metastasis and the growth of malignancies (Pan et al., 2008; Gala and Chan, 2015; Zhao et al., 2016). Previous studies suggest that regular use of aspirin may relieve the severity of the condition in early-diagnosed cancer patients (Langley et al., 2011; Huang et al., 2015). Aspirin regulates the MCF-7 breast cancer cells by inducing apoptotic response and inhibits cell proliferation in a dose- and time-dependent manner (Choi et al., 2013). The correlation between the regular use of aspirin and reduced risk of cancer suggests its chemopreventive and chemotherapeutic properties (Pathi et al., 2012; Jonsson et al., 2013; Tougeron et al., 2014; Maity et al., 2015; Saha et al., 2016). However, free drug aspirin can cause serious gastrointestinal mucosal damage due to its inhibitory effect on cyclooxygenase-1, which is produced in the gastric epithelium cells to protect the inner mucosa layer (Tarnawski et al., 2013). Therefore, safer and more effective cancer therapy requires improved efficiency and lower side effects. Enclosing the target drug in MNPs will increase the drug’s bioavailability and efficacy while targeting the tumor sites, reducing side effects, and ensuring increased survival rates for the cancer patients (Subramanian et al., 2008).

**SCHEME 1:**
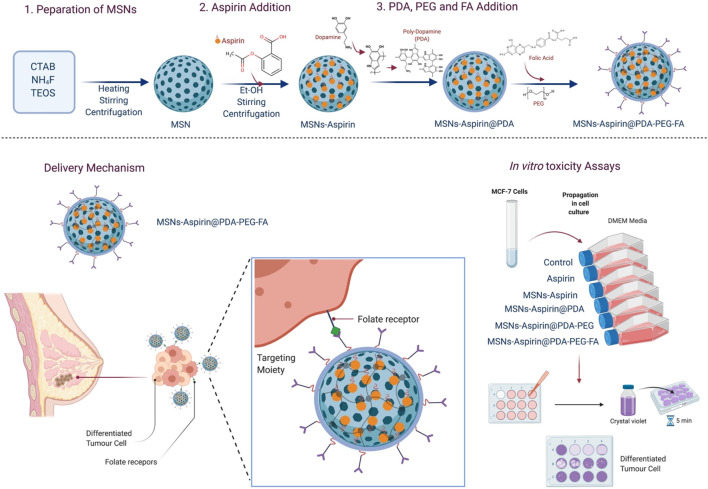
Schematic illustration of the study; synthesis of aspirin-loaded NPs (MNP-Asp@PD-PG-F), delivery mechanism of NPs, and in-vitro toxicity assays.

This study aims to prepare the MNPs based on aspirin in FA-conjugated polydopamine (MNP-Asp-PD-PG-F) delivery vehicle for targeted cancer therapy, particularly for breast cancer treatment. First, MNPs are synthesized as previously reported (Chang et al., 2016; Cheng et al., 2017b). Then, we removed hexadecyltrimethylammonium bromide (CTAB) to increase the pore size and volume. Next, aspirin is loaded into the MNPs by diffusion. We then characterized the size and morphology of aspirin-loaded MNPs using transmission electron microscopy (TEM). The *in vitro* drug release profiles of MNPs at different pH values [phosphate-buffered saline (PBS); pH 7.4, 5.6, and 2.0] are obtained using high-performance liquid chromatography (HPLC) and spectroscopy (Scheme 1). Finally, we tested its antitumor activity against the MCF-7 breast cancer cell line *in vitro*. Our results show a significant effect on the cytotoxicity and inhibition of breast cancer cells.

Overall, this study shows that MNP-Asp-PD-PG and MNP-Asp-PD-PG-F could block breast cancer cell proliferation using the anticancer properties of aspirin. We report that aspirin in FA-derived NPs imparts significant anticancer properties as compared to free drugs. Thus, our findings provide a novel, safe, and powerful nano-medicinal platform for breast cancer treatment.

## Material Methods

MTT [3-(4,5-dimethyl-2-thiazolyl)- 2,5-diphenyl-2H-tetrazolium bromide], CTAB, dimethyl sulfoxide (DMSO), methanol, tetraethylorthosilicate (TEOS), aspirin (Sigma), acetonitrile, mercapto group-terminated PEG-SH, dopamine hydrochloride, and the ammonium fluoride (NH_4_F) reagent and chemicals were purchased from Aladdin Industrial Co., Ltd. (Shanghai, China). Human breast cancer MCF-7 cells were obtained from American Type Culture Collection (ATCC, Rockville, MD, USA).

### MNP Preparation

The MNPs were synthesized using a modified protocol given elsewhere (Chang et al., 2016; Cheng et al., 2017b; Wei et al., 2017). CTAB (1.82 g, 5 mmol) and NH_4_F (3 g, 81 mmol) were dissolved in water, and the mixture was vigorously stirred at 80°C. This followed the addition of TEOS (9 ml, 8.41 g) dropwise. For the next 6 h, the mixture was subjected to vigorous stirring at 80°C in an oil bath. MNPs were collected by centrifugation (10, 000 × *g,* for 12 min) and washed with ethanol/deionized water repeatedly. MNPs were dried overnight with the help of a vacuum. Calcination eliminated the surfactant template (CTAB). The MNPs were gradually heated to 300°C with a rise in temperature by 2°C min^−1^ and then heated to 600°C (1°C min^−1^). The products obtained were kept for 6 h at 600°C (Cheng et al., 2017a).

### MNP Loading With Drug

With the help of diffusion, the aspirin drug was filled in the MNPs. About 100 mg of MNPs were suspended in absolute ethanol (10 ml). Afterwards, 100 mg of aspirin present in the mixture was stirred at room temperature, in a dark space for 24 h, in accordance with the authorized protocols (Lodha et al., 2012; Liu et al., 2016). Centrifugation was used to collect products. As a result, MNPs loaded with aspirin were lyophilized and were termed as MNP-Asp.

### PDA Prime-Coating of MNP

For the coating of core particles with PD, 100 mg particles were suspended in a dopamine hydrochloride solution (50 ml) with a Tris buffer (pH 8.5, 10 mM) at room temperature for 6 h, accompanied with vigorous stirring. The black particles obtained were subjected to centrifugation and washed with water afterwards. Lyophilization was used to dry the NPs with PD coating.

### Addition of PG/PG-F to PD-Coated MNP

The Michael addition reaction resulted in the linkage of functional ligands to PD-coated MNP surface. These PD-coated NPs (100 mg) were added to the Tris buffer (40 ml, pH 8.6, and 20 mM) along with 200 mg ligands (F−PG-SH or G-SH, PG, Mw = 2000), and 2 mg of TCEP. After vigorous stirring for 6 h at 25°C, the nanocarriers (MNP-Asp-PD-PG-F and MNP-Asp-PD-PG) were obtained with the help of centrifugation, and distilled water was used to wash them for the removal of any residual reactants. Final products were subjected to lyophilization.

### Characterization of MNP

Distilled water was used to dilute the dry powder sample, and it was sonicated before the other measurements were performed. TEM (Tecnai G2 F30; FEI Company, Hillsboro, OR, USA) was used to study the images of the surface and shape morphology of NPs. To prepare TEM samples, a specified quantity of particles was suspended in a specified amount of distilled water and sonicated. Prepared MNPs were observed on a carbon-coated Cu grid. At −196°C, the isotherms of N2 adsorption and desorption were recorded using ASAP 2020 accelerated surface area. For 24 h, the MNP sample was degassed by applying vacuum at 120°C. Surface areas were estimated from the data of adsorption along with the method of Brunauer−Emmett−Teller. The sample was subjected to heating at 800°C with a heating ramp of 10°C min^−1^. With the help of HPLC (LC 1200; Agilent Technologies, Santa Clara, CA, USA), the loading content of drug was calculated for aspirin-loaded NPs according to previously authorized methods (Chang et al., 2016). The calibration curves obtained through spectroscopy and HPLC data helped estimate the residual amounts of aspirin in solution.

### Drug Release Profiles

The aspirin release from MNPs was studied using an implied modified method (Zhang and Feng, 2006). The lyophilized sample (5 mg) was dispersed in PBS (1 ml; pH 7.4, 5.6, and 2.0) and dialyzed (cutoff size 3,500 Da) against the sample release medium (10 ml). At 37°C, the tubes were incubated in an orbital shaker water bath (100 rpm). The dialysis buffer was replaced with fresh buffer in equal volumes with predetermined time gaps in the 7-day session. HPLC helped in evaluating the amount of aspirin present in the supernatant.

### *In Vitro* Cytotoxicity

Dulbecco’s modified Eagle medium (DMEM) was used to culture MCF-7 cells, accompanied with antibiotic penicillin (100 IU ml^−1^), fetal bovine serum [10% (v/v)], and streptomycin (100 μg ml^−1^). Cell culture was performed in a humid environment at 37°C with 5% CO_2_−95% air. For next 24–48 h, the cells were taken in 6-well plates. Either free aspirin or its conjugated NPs (containing approx. 5 μg ml^−1^ aspirin) were added to the well plates. The *in vitro* MTT assay helped in detecting the antitumor activity of NPs toward MCF-7 cells (Chang et al., 2016). 96-well culture plates were used to seed MCF-7 cells containing 8 × 10^3^ cells per well. Then, the cells were subjected to various amounts of free aspirin or aspirin-conjugated NPs (approx. 0.125–2.5 μg ml^−1^ aspirin) for the next 24 and 48 h. A stock solution [10 μl of MTT (5 mg ml^−1^)] was added to each well. It was further incubated for 4 h. The medium was removed afterwards, and the formazan crystals formed inside the living cells were solubilized by means of the DMSO amount of 100 μl per well. It was subjected to 10 min of gentle shaking, and the absorbance value was obtained at 590 nm wavelength on a microplate reader (Bio-Rad Model680, United Kingdom). The cell values without drugs and MTT were assigned as 1 and 0, respectively, to get the normalized absorbance value.

### Breast Cancer Cell Proliferation Assay

Breast cancer cell lines were grown in 6-well plates after treatment with aspirin or modified NPs for 72 h, and then cells were fixed and stained with crystal violet. To quantify staining, acetic acid (1 ml, 10%) was added to extract dye in each well, and the absorbance value was obtained at 590 nm with a reference wavelength at 750 nm (Wang et al., 2015).

### MNP Uptake Assay

The relative uptake of the MNPs (MNP-Asp-PD-PG and MNP-Asp-PD-PG-F) was also monitored semi-quantitatively while measuring aspirin contents in the MCF-7 cells by using HPLC (LC 1200; Agilent Technologies, Santa Clara, CA, USA). The MCF-7 cells were seeded into 6-well plates as 8 × 10^3^ cells per well. Then, the cells were subjected to MNP-Asp-PD-PG and MNP-Asp-PD-PG-F (approx. 100 μg ml^−1^) for the next 24 h at 37°C. The cells were washed with three times with ice-cold PBS and trypsinized for 15 min, and media fractions were centrifuged (14, 000 × g), followed by cell pellet lysis by a lysis buffer containing 50 mM Tris, pH 7.5; 200 mM NaCl; 1 mM EDTA, and 0.1% tween 20. The cell lysate was analyzed by HPLC as described above.

### Statistical Analysis

All the experiments were repeated at least three-to-five times. The experimental data were expressed as the mean ± standard deviation. Statistical analysis was performed by two-way ANOVA, followed by the graph pad prism. *p < 0.05 represents statistical significance, and **p < 0.01 represents extreme statistical significance.

## Results and Discussion

### Synthesis and Characterization of MNP-Asp-PD-PG-F

**FIGURE 1:**
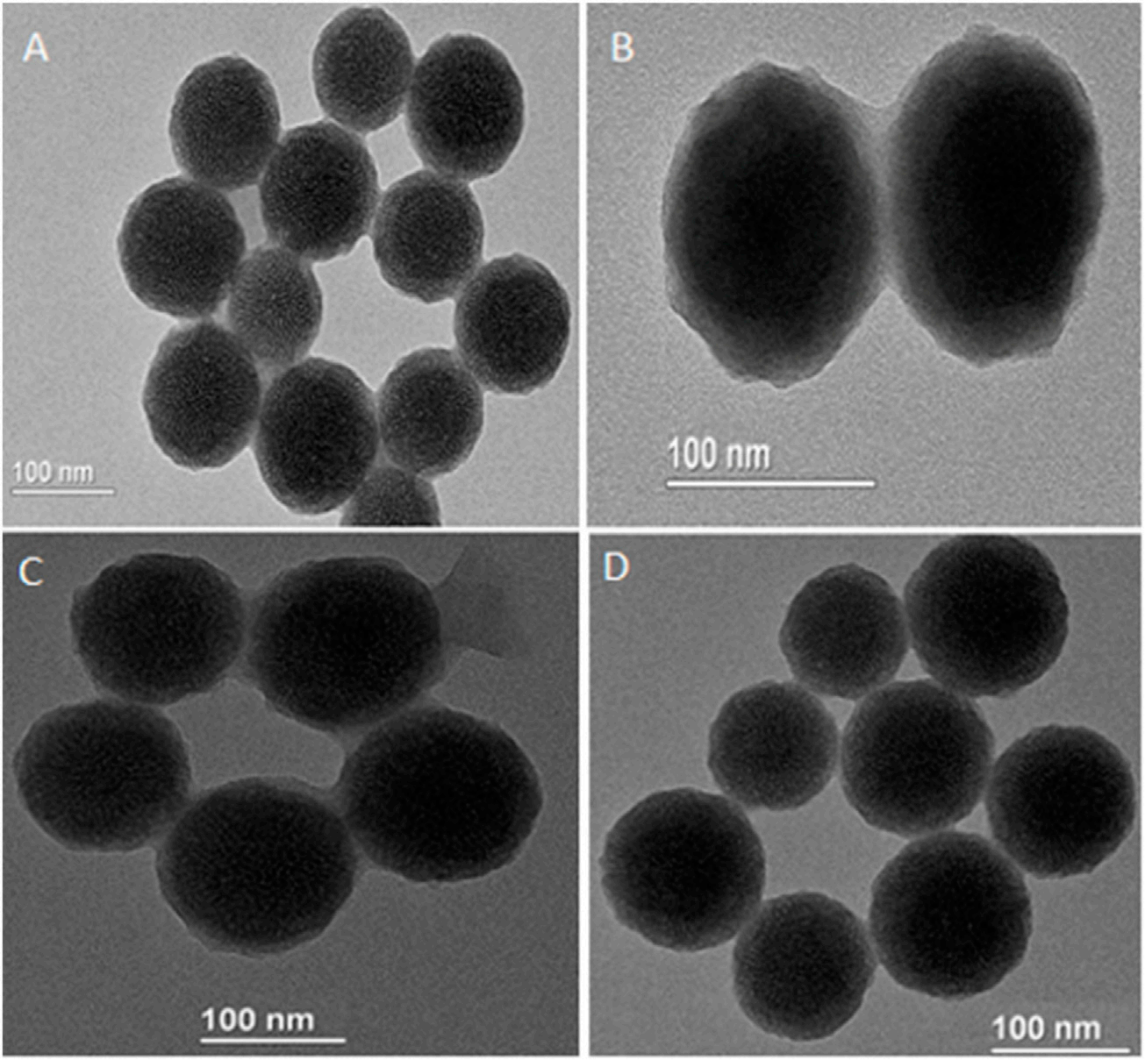
TEM images of **(A)** MNPs-aspirin particles (size 147.00 ± 6.0 nm) **(B)** MNP-Asp@PD particles (size 160.7 ± 8.3) **(C)** MNP-Asp@PD-PG particles (size 197.4 ± 6.4), and **(D)** MNP-Asp@PD-PG-F particles (size 204.4 ± 6.0).

Scheme 1 illustrates a four-step preparation of MNP-Asp-PD-PG-F MPs as described in *Methods*. Next, we characterized MNPs by using TEM (Table 1). The obtained size distribution displayed a dynamic range of fictitious NPs about 100–200 nm in diameter that is in line with the EPR (enhanced permeability and retention) effect theory for more penetration and cellular uptake of NPs into tumors (Win and Feng, 2005; Zhao and Feng, 2014; Kalyane et al., 2019). Thus, the obtained larger size and shape of our synthesized MNPs ensure no extravasation in normal tissues, while there is a maximum retention of administered nano-formulation in tumors with reduced adverse effects. The TEM images of MNP-Asp in Figure 1A show that MNP-Asp nanoparticles were slightly rough and porous, while the obtained average size was about 147.00 ± 6.0 nm.

**TABLE 1:** Characterization of prepared Nanoparticles.

	Polymers	Size (nm)
1	MNPs	139.00 ± 6.0
2	MNPs-Asp	147.00 ± 6.0
3	MNPs-Asp-PD	167.70 ± 8.3
4	MNPs-Asp-PD-PG	197.40 ± 6.4
5	MNPs-Asp-PD-PG-FA	204.40 ± 6.0

Our MNPs appear chemically and thermally stable with clear, controllable morphology and porosity. Moreover, to reduce the vascular irritation of the loaded drug and improve its water solubility, tumor targeting, and bioavailability (Zhang et al., 2019; Zha et al., 2020), MNP-Asp was further coated with polydopamine (PDA) to develop MNP-Asp-PDNPs through anti-solvent precipitation and surface modification. We obtained the average size of the MNP-Asp-PD, which is 160.7 ± 8.3 nm, as shown by the TEM image of MNP-Asp-PD in Figure 1B. The coating increased the particle size of MNP-Asp-PD by 13 nm compared to MNP@Asp, suggesting the successful coating of PDA.

Then, to render the NPs with hydrophilic and antifouling properties, we coated the MNP-Asp-PDNPs with PEG. TEM images show the resulting MNP-Asp-PD-PG, NPs with a size of 197.4 ± 6.4 nm in Figure 1C. The PEGylated particles can provide various positive influences such as preventing particle aggregation in water, maximizing the particle circulation in the bloodstream and their relativity (Patsula et al., 2019; Lazaro-Carrillo et al., 2020).

Since cancer cells tend to express several FRs on their surface, the FA-modified nanoparticles can facilitate a rapid internalization of drug molecules inside the cancer cells (Ansari et al., 2020). Therefore, we modified the MNP-Asp-PD-PG by coating F, and the resulting particles were designated as MNP-Asp-PD-PG-F. The MNP-Asp-PD-PG-F particle size was found to be 204.4 ± 6.0 nm, as shown through TEM (Figure 1D). The morphology of all NPs appears as uniform and monodispersed spheres of high porosity through TEM images, as shown in Figures 1A–D. The hexagonal mesopore’s diameter was around 2–3 nm. TEM also shows a rough shell around the NPs of MNP-Asp-PD and MNP-Asp-PD-PG-F, confirming the PD film formation.

### *In Vitro* Drug Release Kinetics

**FIGURE 2:**
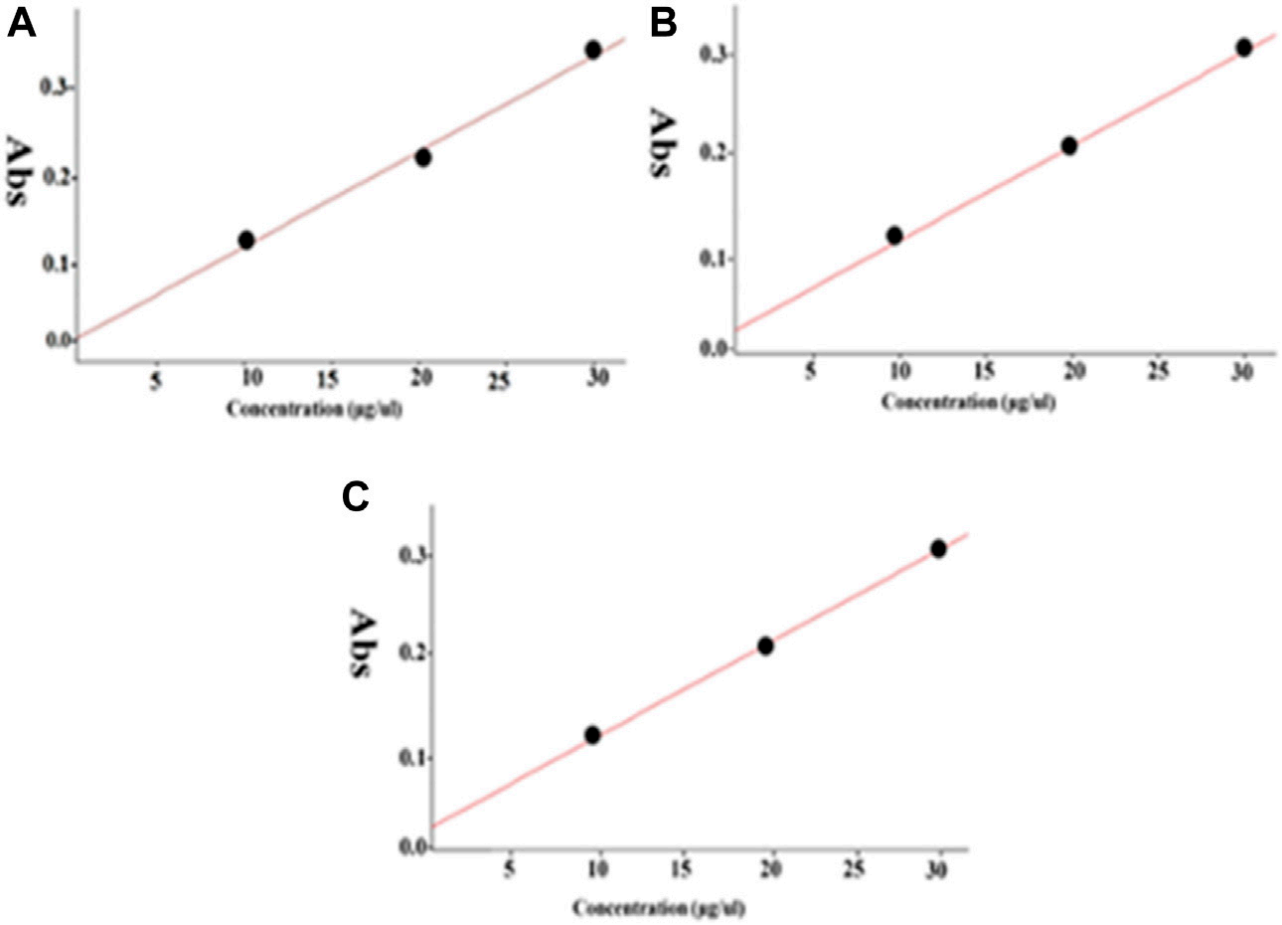
The spectra for *in vitro* drug release through MNP-Asp@PD-PG-F with different pH media. **(A)** The pH value 7.4, **(B)** 5.6, and **(C)** 2.0.3.3.

Herein, we studied the release of drug from MNP-Asp-PD-PG-F. Drug release with respect to various pH values including 7.4 (Figure 2A), 5.6 (Figure 2B), and 2.0 (Figure 2C) was observed at 37°C. A typical biphasic drug release pattern was observed for all of the NPs with FA showing an initial (24 h) aspirin burst release, which was later sustained up to 190 h (Figure 2).

The MNP-Asp-PD−PEG−FA exhibited a control release rate under all tested pH values. The characteristic release pattern of the drug from FA-derived nanoparticles suggests that the PD coating may block the MNPs’ pores and provide effectually controlled drug release. For MNP-Asp-PD−PG−F, the drug release rate increased with elevated time intervals at acidic or basic pH (Figure 2). The results agree with previous reports that the PDA-coated particles at physiological pH could provide retained drug diffusion and sustained release over a more extended period of time while essentially preserving the drug delivery system’s structure. This prolonged transmission probably results from intermolecular interactions between the active drug and functional decorations of NPs (Lim et al., 2020).

**FIGURE 3:**
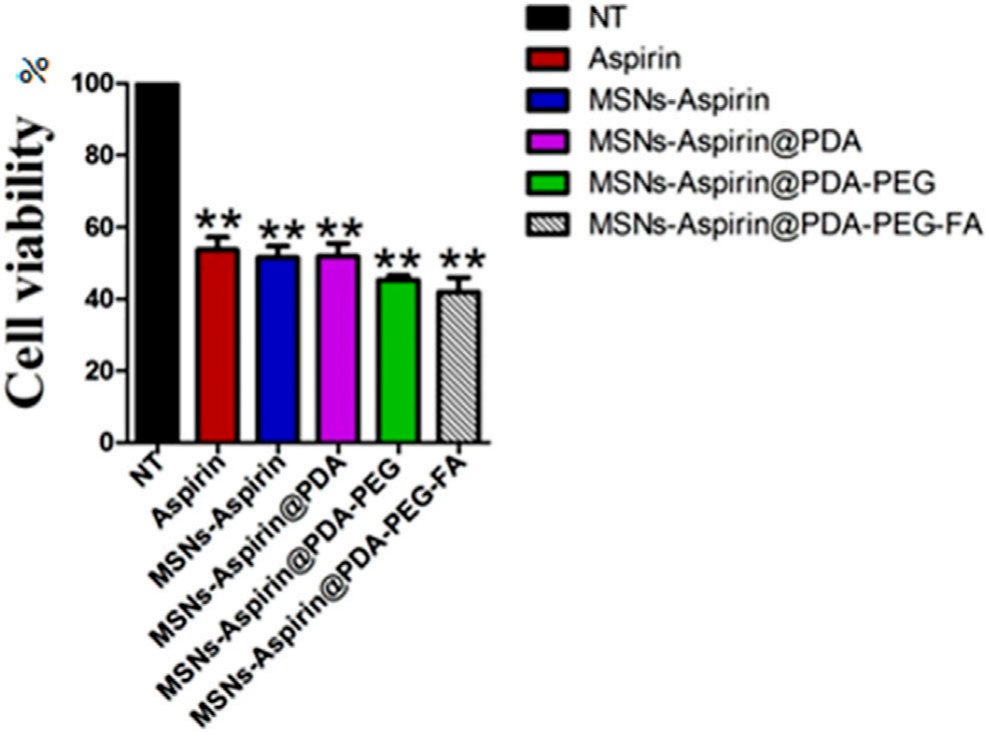
MTT assay based cells viability of MCF-7 breast cancer cells examined at 24 h; Cell culture is treated with Negative control (NT), free drug (Aspirin), drug-loaded in MSNs (MNP-Asp), and all other derivatives of Aspirin loaded MNPs prepared in this study (MNP-Asp@PD, MNP-Asp@PD-PG, and MSNs-Asp@PD-PG-F).

To overcome aspirin’s gastrointestinal mucosal damage (Gao et al., 2019; Tang et al., 2020), MNPs were loaded with aspirin to treat breast cancer and the cytotoxic effects were examined while comparing aspirin and aspirin-loaded MNPs through the MTT assay. We observed that aspirin-loaded MNPs (MNP-Asp-PD-PG-F) show 15–25% stronger cytotoxic effects against cancer cells than the free drug (Figure 3). Moreover, this result confirms that synthesized NPs are biocompatible with no observed toxicity toward normal cells and tissues. Our findings agree with parallel studies (Tran et al., 2019) that MNP-Asp-PD-PG-F NPs hold increased cytotoxic and anticancer effects compared to other drug-loaded NP derivatives in this study.

**FIGURE 4:**
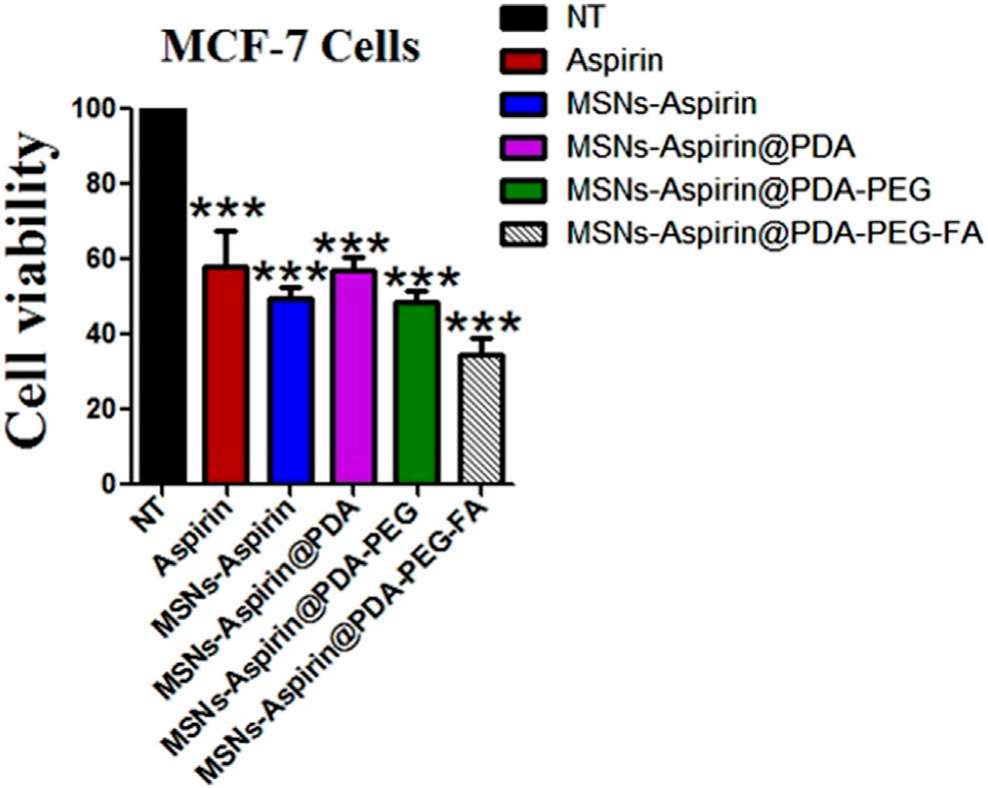
MTT assay based cells viability of MCF-7 breast cancer cells examined at 48 h; Cell culture is treated with Negative control (NT), free drug (Aspirin), drug-loaded in MNPs (MSNs-Aspirin), and all other derivatives of Aspirin loaded MNPs prepared in this study (MNP-Asp@PD, MNP-Asp@PD-PG, and MNP-Asp@PD-PG-F).

In addition, we noticed that increasing the incubation time of the MNP-Asp-PD-PG-F derivative lowered cells’ viability to <40%, indicating a time-dependent cytotoxic effect that, in turn, shows the enhanced therapeutic effect through this platform based on aspirin in FA-conjugated polydopamine-modified MNPs on the breast cancer MCF-7 cell line (Figure 4). Aspirin and aspirin-loaded/modified MNPs, when applied to HK cells, showed very low toxicity (Supplementary Figure S1). Altogether, these results suggest that the MNP-Asp-PD-PG-F inhibits *in vitro* cell growth; hence, it could be an attractive direction to the therapeutic strategy of breast cancer.

### Free Drug Aspirin or Modified MNP Inhibits the Proliferation Ability of the Breast Cancer Cells

**FIGURE 5:**
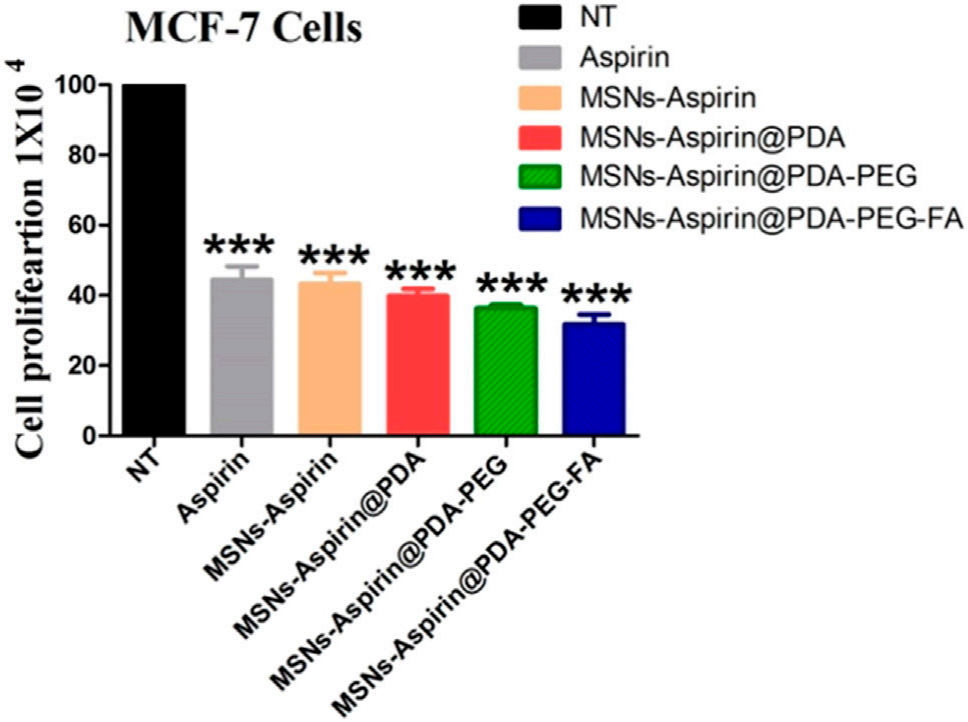
Proliferation assay of MCF-7 breast cancer cells examined at 72 h; cell culture is treated with negative control (NT), free drug (aspirin), drug-loaded in MNPs (MNP-Asp), and all other derivatives of aspirin-loaded MNPs prepared in this study (MNP-Asp@PD, MNP-Asp@PD-PG, and MNP-Asp@PD-PG-F).

We also examined the proliferation of breast cancer cells in the presence of the free drug (aspirin) and all the derivatives (MNP-Asp, MNP-Asp-PD, MNP-Asp-PD-PG, and MNP-Asp-PD-PG-F) with equal concentrations and for the same incubation interval (Figure 5).

The results show that MNP-Asp-PD-PG-F increased the inhibition of cancer cell proliferation (around 20%) compared to the free drug or other derivatives. Our results agree with an earlier report in which MSN-quercetin@FA NPs reduce MDA-MB 231 cell viability by arresting the cell cycle and inducing apoptosis (Sarkar et al., 2016). Aspirin inhibits protein synthesis initiation (Kwon et al., 1997), COX2 activities (Kashfi and Rigas, 2005), and TGF-β1-induced COX-2 expression (Zhao et al., 2018). It has also been reported to activate caspases (Dikshit et al., 2006), the ceramide pathway (Chan et al., 1998), the tumor suppressor gene (Mahdi et al., 2006), and oxidative stress (Dikshit et al., 2006).

**FIGURE 6:**
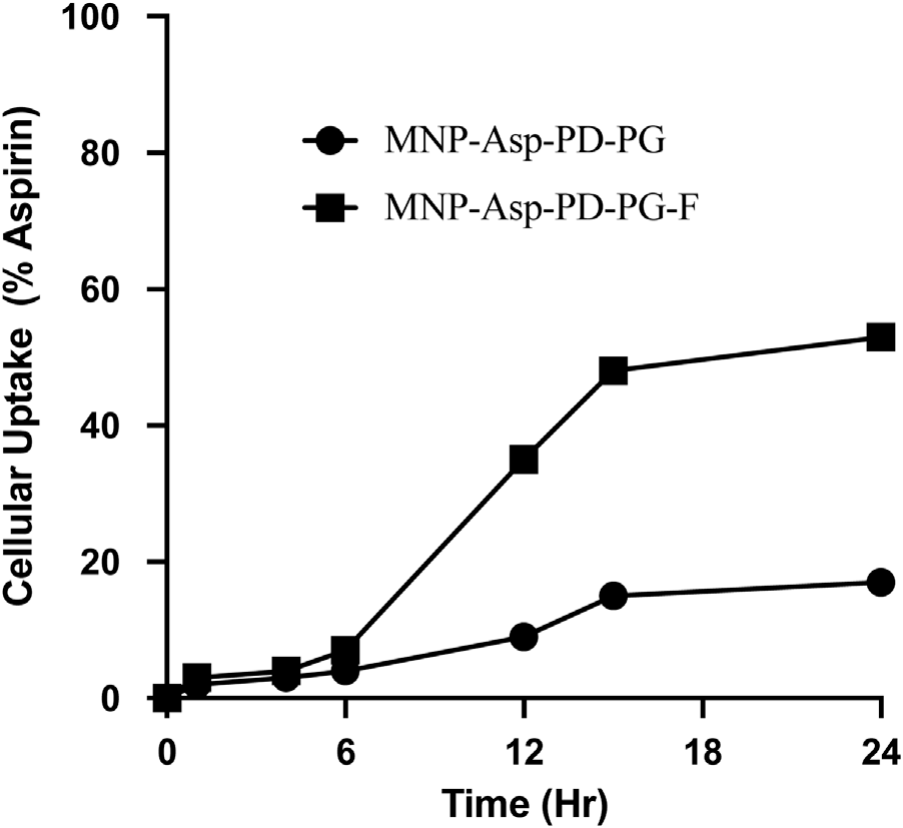
Cellular uptake assay of MCF-7 breast cancer cells examined at 2, 4, 6, 12, 15, and 24 h; cell culture is treated with drug-loaded in Aspirin loaded MSNs prepared in this study (MNP-Asp@PD-PG and MNP-Asp@PD-PG-F).

In the MNPs’ uptake assay, the relative concentration of aspirin in the cell was monitored in the MCF-7 cells. The aspirin concentration in the cell after 24 h was found to be 17% from the cell lysate when given in the form of MNP-Asp-PD-PG, while it was observed as 53% when MNP-Asp-PD-PG-F MNPs were used (Figure 6). This shows that folate plays an important role in the targeted delivery of the aspirin to the breast cancer cell lines. For future studies, the fluorescence and confocal microscopy studies are recommended to complete the elucidation of MNPs’ entry in the cancer as well as in normal cells.

## Conclusion

This study synthesized FA-decorated, PD-modified NPs (MNP-Asp-PD-PG-F) for the sustained and controlled delivery of aspirin for the targeted therapy of breast cancer. MNP-Asp-PD-PG-F possesses a diameter of about 204.4 ± 6.0 nm and high levels of drug LC. TEM provided a clear image of PDA film coating on the NPs’ surface. The high pH sensitivity of MNP-Asp-PD-PG-F was detected through the in vitro profiles of the drugs released. MNP-Asp-PD−PG−F showed high cytotoxicity than that of free aspirin or its other modified NPs. We also noticed that the release of a drug from NPs slightly depends on the acidic environment. Moreover, the MNP-Asp-PD-PG-F significantly blocked breast cancer cell proliferation compared to free aspirin or other supplementary modified NPs, suggesting the MNP-PD-PG-F as a promising nanocarrier of various antitumor drugs used for cancer treatment.

## Data Availability

The original contributions presented in the study are included in the article/Supplementary Material, further inquiries can be directed to the corresponding authors.

## Supplementary Material

Supplementary Figure 1 MTT assay-based cell viability of human kidney (HK) cells examined at 48 h; cell culture is treated with negative control (NT), free drug (aspirin), drug loaded in MSNs (MSNs aspirin), and all other derivatives of aspirin loaded MSNs prepared in this study (MNP-Asp@PD, MNP-Asp@PD-PG, and MNP-Asp@PD-PG-F).
