# Supplementary material for: Aspirin Repurposing in Folate-Decorated Nanoparticles: Another Way to Target Breast Cancer
Source: Front Mol Biosci. 2022 Jan 28;8:788279. doi: 10.3389/fmolb.2021.788279 (PMC8848101; doi:10.3389/fmolb.2021.788279)
Supplement: Supplementary file 1 [file Presentation1.pdf]

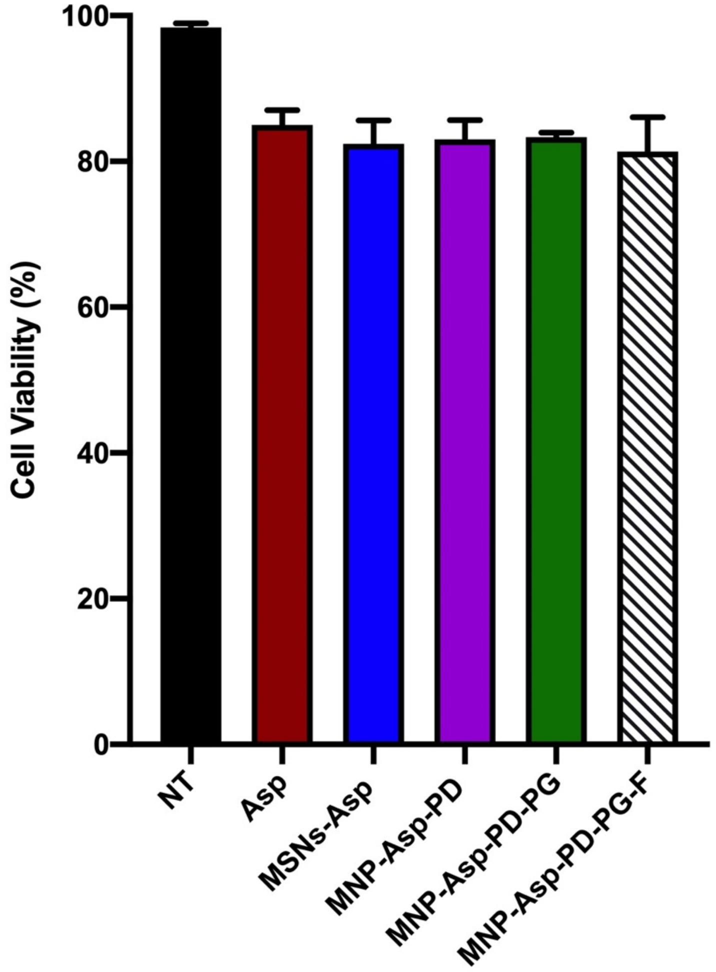

Figure S1: *MTT assay based cells viability of Human Kidney (HK) cells examined at 48 h; Cell culture is treated with Negative control (NT), free drug (Aspirin), drug-loaded in MSNs (MSNs-Aspirin), and all other derivatives of Aspirin loaded MSNs prepared in this study (MNP-Asp-PD, MNP-Asp-PD-PG, MNP-Asp-PD-PG-F).*
